# Evaluation of the bronchial arteries: normal findings, hypertrophy and embolization in patients with hemoptysis

**DOI:** 10.1186/s13244-020-00877-4

**Published:** 2020-05-19

**Authors:** João Almeida, Cecília Leal, Luísa Figueiredo

**Affiliations:** Department of Radiology, Hospital de Santa Marta, Rua de Santa Marta, 1169-024 Lisbon, Portugal

**Keywords:** Bronchial arteries, Hemoptysis, Embolization, Computer tomography, Angiography

## Abstract

The enlargement of the bronchial arteries occurs in a multitude of congenital and acquired diseases and is responsible for the majority of cases of hemoptysis. In this review, we provide a simplified imaging approach to the evaluation of the bronchial arteries. We highlight the anatomy and function of the bronchial arteries, typical imaging findings, how to recognize bronchial artery dilatation, and its underlying causes. Contrast-enhanced computer tomography plays a major role in diagnosing bronchial artery enlargement and also improves treatment planning. Bronchial artery embolization has proven to be effective in controlling the potential hazardous hemoptysis.

## Key points

- Bronchial arteries most commonly originate from the descending thoracic aorta, although they may also have an ectopic origin from nearby arteries.
- Normal bronchial arteries are very thin (< 1.5 mm).
- The causes of bronchial artery dilatation are variable and include congenital and acquired diseases.
- Bronchial artery enlargement can be recognized when its caliber exceeds 2 mm, increasing the risk of clinically significant hemoptysis.
- Embolization is an effective technique in the management of patients with hemoptysis.

## Introduction

The lungs have a dual vascular supply—pulmonary and bronchial.

The pulmonary arteries have a major role in gas exchange, delivering deoxygenated blood to the lungs. The bronchial arteries have a supportive role, with a much thinner caliber than the pulmonary arteries, and they carry oxygenated blood to the bronchial tree, large blood vessels, lymph nodes, esophagus, and pleura [1]. The bronchial arteries have distal microvascular anastomoses connecting to the pulmonary arterial system [2].

Most of the time, in healthy patients, the bronchial arteries are very thin and difficult to detect on contrast-enhanced computer tomography (CECT) examinations. Some diseases may lead to an impairment of the pulmonary circulation. Bronchial arteries have a notable plasticity, potentially increasing its flow from 1 up to 30% of the cardiac output in response to a pulmonary insult. Consequently, bronchial artery hypertrophy (BAH) and dilatation of the thin-walled distal bronchial-to-pulmonary artery anastomosis may occur. However, this recruitment increases the risk of bronchial artery rupture with subsequent pulmonary hemorrhage [1]. Bronchial arterial system is the main source of bleeding in 90% of the cases of massive hemoptysis, followed by the pulmonary arteries (5%), and the non-bronchial systemic arteries (5%) [3].

CECT is valuable in patients with hemoptysis, allowing the visualization of dilated bronchial or non-bronchial arteries potentially responsible for hemoptysis, providing a roadmap to guide the interventional approach.

The aim of this pictorial review is to familiarize the reader with the normal anatomy of the bronchial arteries and its most common variants, how to recognize BAH, underlying diseases, and finally, to discuss the role of bronchial artery embolization in patients with hemoptysis.

## Anatomy of bronchial arteries

**Fig. 1:**
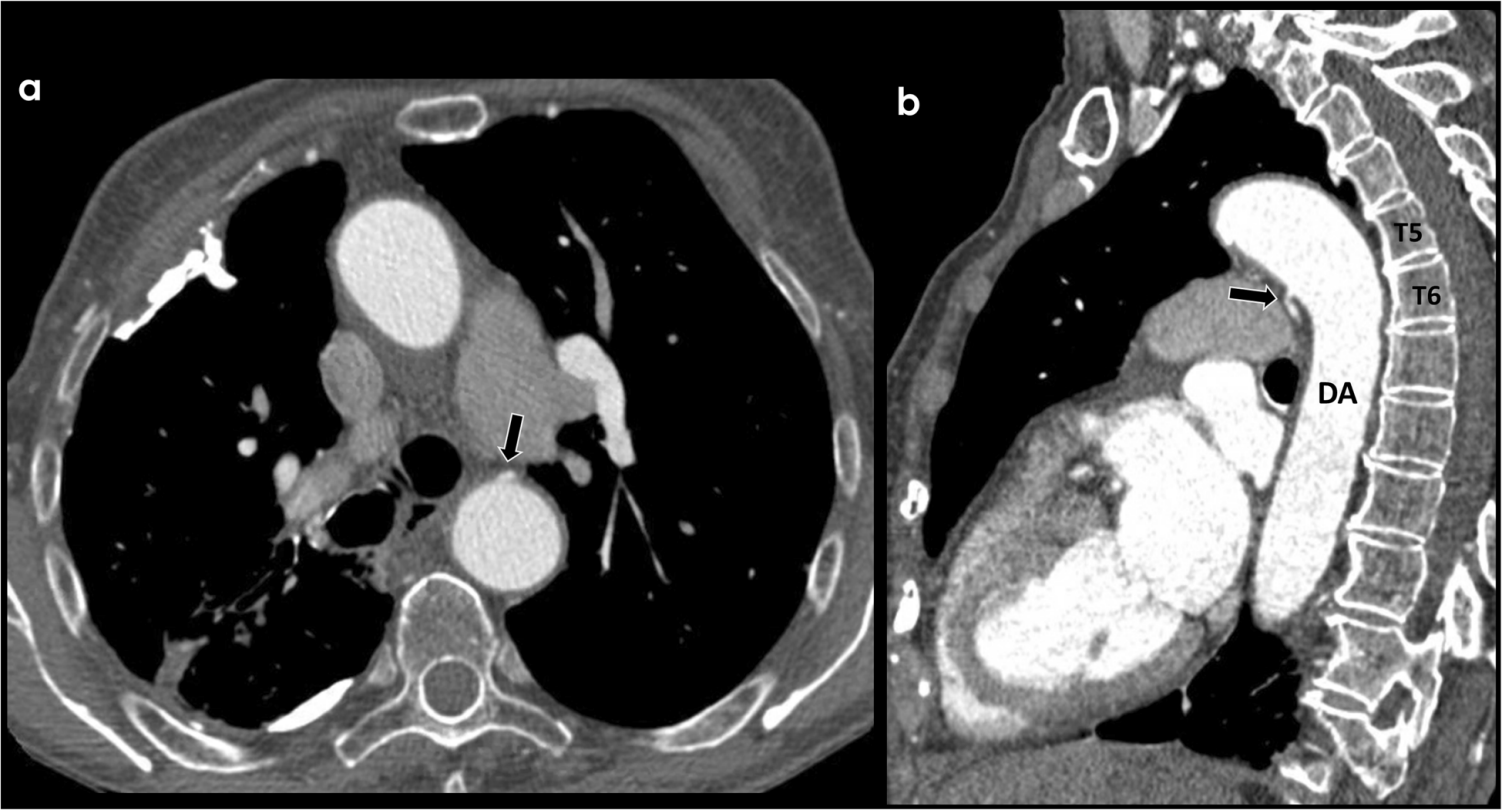
Origin of orthotopic bronchial arteries. Axial (**a**) and sagital (**b**) CECT view of an orthotopic bronchial artery (black arrows) originating from the descending thoracic aorta (DA) at the T5–T6 vertebral plane, near the carinal level

The anatomy of the bronchial arteries is quite variable in terms of origin and number. Normal bronchial arteries are small vessels, most commonly originating from the descending thoracic aorta (orthotopic), usually at the T5-T6 vertebral plane, 1–2 cm above or below the carinal level (Fig. 1).

In up to 36% of cases, they can originate elsewhere (ectopic), such as from the aortic arch, subclavian artery, thyrocervical trunk, internal mammary artery, and coronary arteries [4–7]. Ectopic bronchial arteries are recognized due to its adjacent course with the associated bronchi.

In its orthotopic origin, the right bronchial arteries preferentially arise from the posteromedial aortic wall, directly or more commonly (67% of cases) from a short intercostal-bronchial trunk (ICBT). The left bronchial arteries typically originate from the anterior or lateral aortic wall, both running towards each hilum with a retrotracheal course [8].

**Fig. 2:**
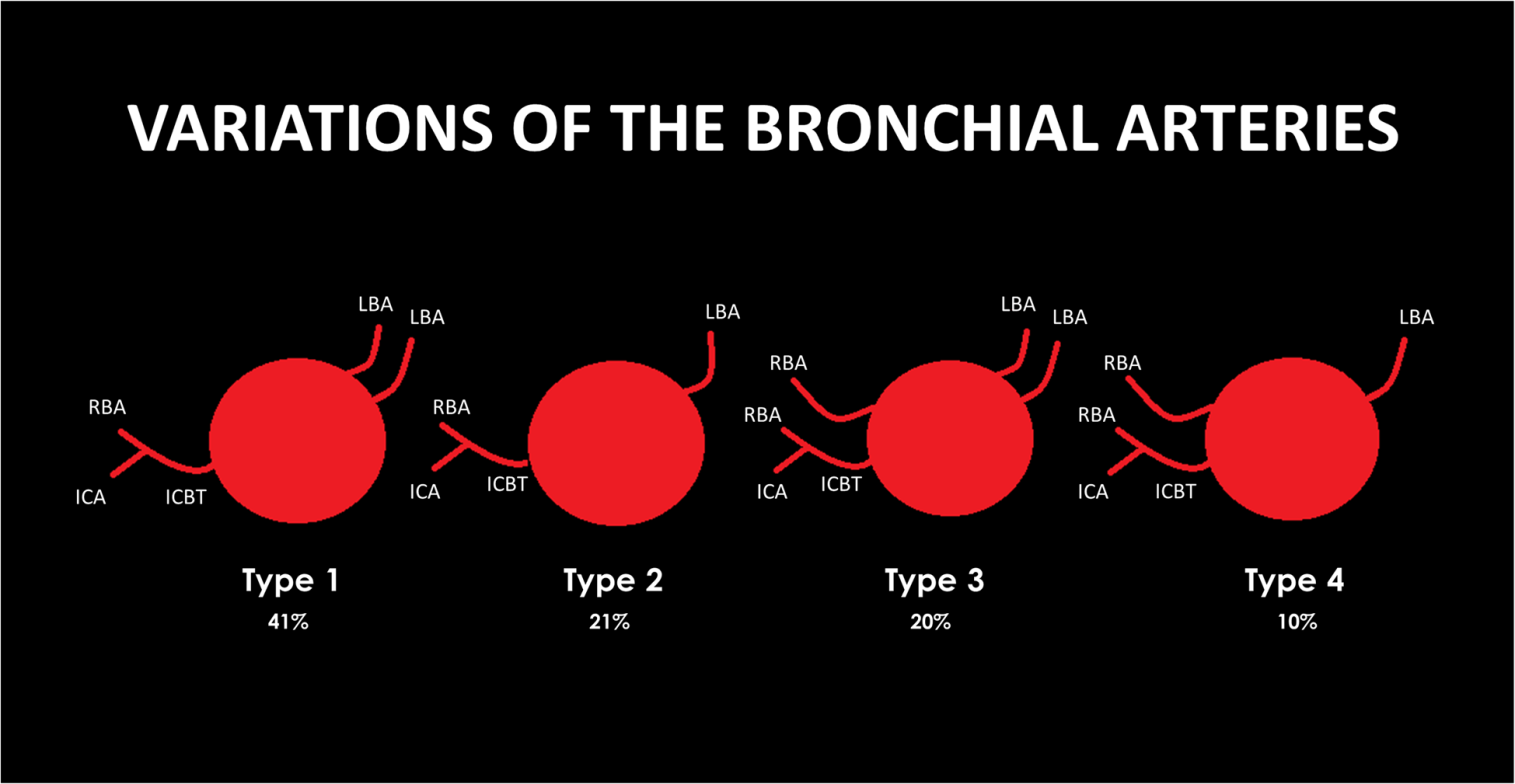
Variations of the bronchial arteries. Bronchial arteries may have a great variability in terms of number. The most common patterns are two left and one right bronchial artery (type 1, most common), one artery in each side (type 2), two right and two left bronchial arteries (type 3), and two right and one left bronchial artery (type 4). Usually, the right bronchial artery originates from an intercostal-bronchial trunk (ICBT). RBA, right bronchial artery; LBA, left bronchial artery; ICBT, intercostal-bronchial trunk; ICA, intercostal artery

Cauldwell et al. described several variations in terms of the number and origin of the bronchial arteries, although four branching types were notably more prevalent (Fig. 2). The most common (type 1) is two left and one right bronchial artery, the latter originating from an ICBT, with a reported incidence of 41% [8, 9]. Other common variants include one artery in each side, the right bronchial artery originating from an ICBT (type 2), two right and two left bronchial arteries (type 3), and two right and one left bronchial arteries (type 4), respectively with 21%, 20%, and 10% of occurrence [9]. The normal caliber of the bronchial arteries is less than 1.5 mm near the origin and less than 0.5 mm distally, as they ramify in the pulmonary hila. When hypertrophy occurs, its diameter usually exceeds 2 mm, and they tend to have a more tortuous course. Venous bronchial drainage is mainly through the pulmonary veins, with a smaller contribution of drainage through the superior vena cava, azygos, and hemyazigos systems [10].

## Imaging of bronchial arteries

CECT frequently allows identification of the bronchial arteries and non-bronchial systemic arteries, particularly with modern scanners due to higher spatial resolution. In the evaluation of the bronchial arteries, CECT imaging should be acquired from the supraclavicular regions to the level of the renal arteries, depicting both orthotopic and ectopic bronchial arteries and possible collateral branches to the pulmonary arterial system.

For improved assessment, CECT protocol should be optimized using high volume and high flow, usually with intravenous injection of 100–120 mL (1.2 mL/kg) of non-ionic contrast medium (350 mg/mL), at a rate of 3–4 mL/s via an antecubital vein. The acquisition benefits from a bolus-tracking technique, aiming for the higher enhancing phase of the descending thoracic aorta and the bronchial circulation (threshold of 120 HU). The imaging parameters are scanner-dependent, frequently with a beam width of 10 mm, beam pitch of 1.2–1.5, tube voltage of 80–120 kV, and a tube current of 90–140 mA (Table 1).

**Table 1:** Multi-detector row CT parameters

Parameters - Multi-detector row CT for bronchial artery evaluation	
Volume of interest	Supraclavicular regions to L2 (level of renal arteries)
Tube voltage	80–120 kV
Tube current	90–140 mA
Collimation	64 × 0.6 mm
Pitch	1.2–1.5
Contrast material	
Volume	100–120 mL
Concentration	350 mg/mL
Injection rate	3–4 mL/s

Suggested multi-detector row CT parameters for bronchial artery evaluation

Cardiac gating and iterative model reconstruction can be useful in the reduction of motion artifacts and noise [11]. Dual-energy spectral CT with low kiloelectron volt monoenergetic reconstructions may also improve image quality, providing the best contrast-to-noise ratio for depicting the bronchial arteries [12].

**Fig. 3:**
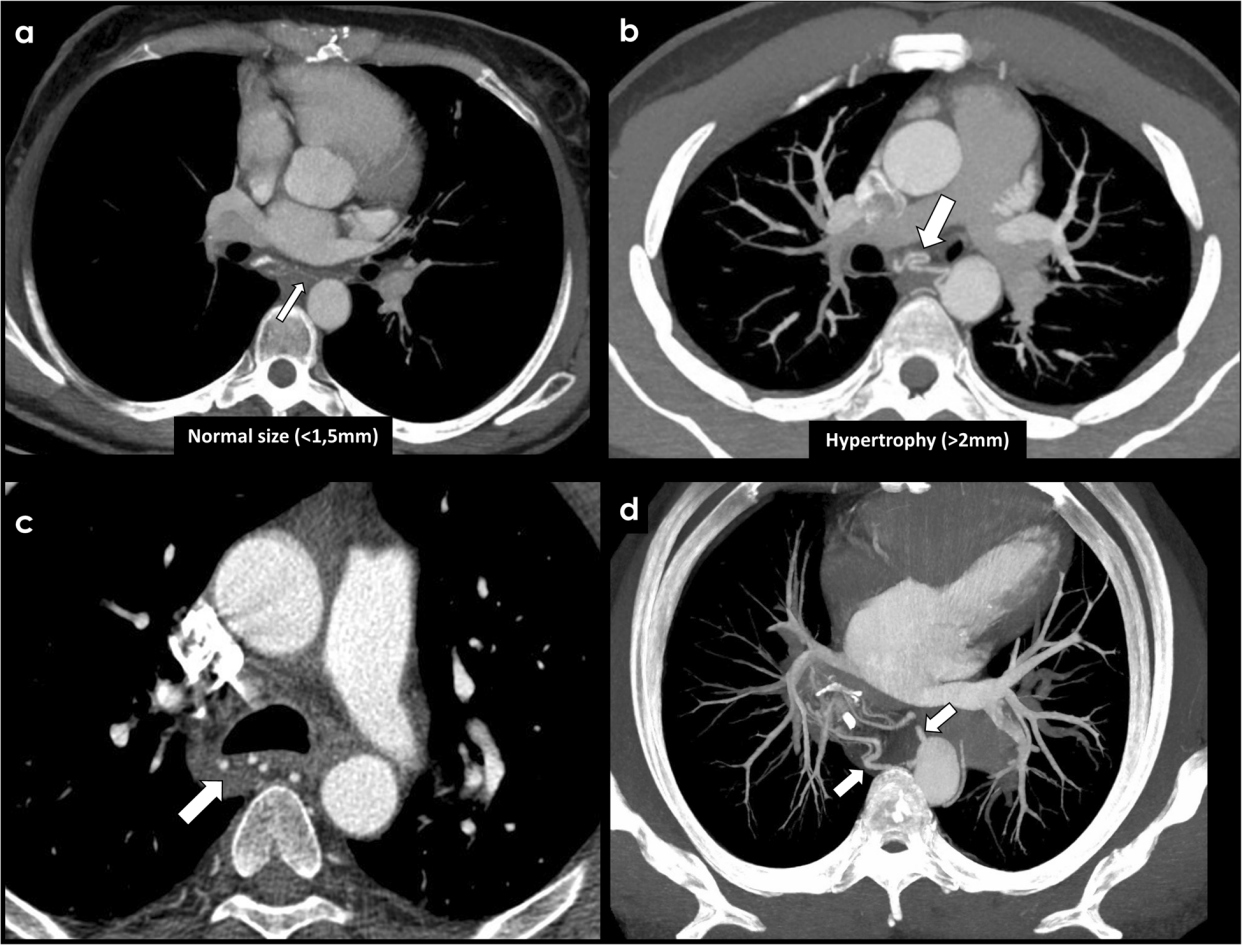
Imaging of the bronchial arteries. CECT findings of normal bronchial arteries (**a**, arrow), with a reduced caliber inferior to 1,5 mm. The remaining images show a patient with hypertrophy of the bronchial arteries, with a caliber exceeding 2 mm (**b**, **c**, **d**; arrows). MIP images (**b**, **d**) are useful when depicting small vessels like the bronchial arteries, displaying its tortuous course in a single image. The patient had a long-term history of bronchiectasis

A low-frequency reconstruction algorithm is preferred when assessing the bronchial arteries, increasing its conspicuity. The images should be reconstructed preferably with thin slices (≤ 1.25 mm), to avoid partial volume effect, and visualized in multiplanar reformats due to the tortuosity of the bronchial arteries [4, 10, 13]. Interactive maximum intensity projection (MIP) is a volume-rendering technique that can be useful when depicting small vessels like the bronchial arteries, displaying its tortuous course in a single image (Fig. 3b, d), being complementary to the axial images. Section thickness and obliquity of the reformatted images should be adjusted in each patient to accurately depict the involved vessels.

Normal bronchial arteries have a reduced caliber (< 1.5 mm) at their origin (Fig. 3a). At CECT, they appear as linear enhancing structures with an ondulating or tortuous morphology in the mediastinum between its orthotopic origin and the retroesophageal/retrotracheal region (Fig. 3a). Sometimes, there are structures that can mimic bronchial arteries due to the proximity of its origin, especially in non-enhanced CT exams, such as regional lymph nodes or azygos vein. Axial images are essential to the initial approach and identification of the origin of bronchial arteries, and should be complemented with assessment of multiplanar and MIP images, particularly when evaluating the mediastinal or hilar tortuous course of the bronchial arteries.

Bronchial arteries larger than 2 mm are typically considered abnormally enlarged or hypertrophied, with an increased risk of bleeding (Fig. 3b, c, d) [7, 8, 14]. Even when their caliber is less than 2 mm, its traceability from the origin to the hilum is associated with bronchial arteries potentially causing hemoptysis [14]. Extravasation of contrast medium is a direct sign of bronchial hemorrhage, although it is unfrequently seen, with reported prevalence of 4–11% [15].

## Causes of bronchial artery hypertrophy

There is a multitude of congenital and acquired diseases that can lead to BAH. These include lung parenchymal diseases, airway-centered diseases, or pulmonary vascular diseases, which directly or indirectly cause a decrease in pulmonary blood flow, leading to chronic pulmonary ischemia. In response, the bronchial arterial system reacts with compensatory recruitment and hypertrophy, increasing lung perfusion through distal anastomoses with the pulmonary arterial system [2, 16].

In patients with BAH, it is important to understand the underlying cause [10]. We will briefly review the major causes of bronchial artery dilatation (Table 2) in the following sections.

**Table 2:** Causes of BAH

Congenital diseases	Acquired diseases
Tetralogy of Fallot	Bronchiectasis
Pulmonary agenesis	Cystic fibrosis
ALCAPA syndrome	Tuberculosis
	Aspergilloma
	Lung abscess
	Chronic thromboembolic disease
	Pulmonary hypertension
	Takayasu arteritis
	Fibrosing mediastinitis
	Malignancy

List of the most common congenital and acquired diseases associated with BAH

### Congenital causes of BAH

The most common congenital diseases associated with bronchial artery dilatation are Tetralogy of Fallot, pulmonary agenesis, and an anomalous left coronary artery arising from the pulmonary artery (ALCAPA syndrome).

**Fig. 4:**
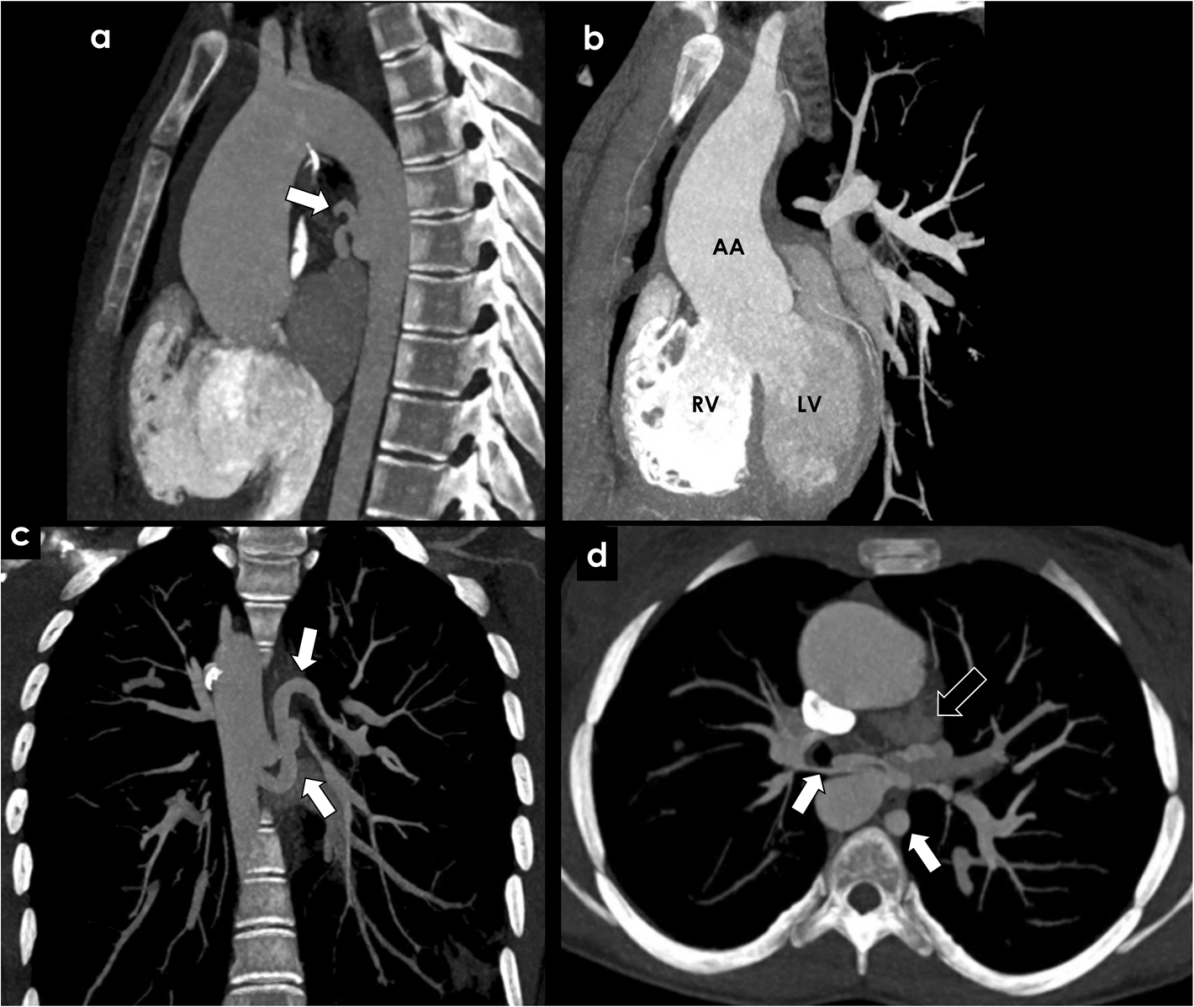
Congenital causes of BAH—Tetralogy of Fallot and pulmonary agenesis. Significant bronchial artery hypertrophy (white arrows) in a patient with tetralogy of Fallot (**a**–**c**) and in a patient with pulmonary artery agenesis (**d**)—“image **b**” shows a ventricular septal defect, overriding aorta and right ventricular hypertrophy, features of tetralogy of Fallot. The black arrow in “image **d**” depicts the absence of the pulmonary artery. RV, right ventricle; LV, left ventricle; AA, ascending aorta

Tetralogy of Fallot is the most common cyanotic congenital heart condition. Classically, it is characterized by the combination of right ventricular outflow tract obstruction, ventricular septal defect, overriding aorta, and right ventricular hypertrophy [17]. When severe, it is associated with pulmonary valve atresia, leading to an impairment of the pulmonary arterial flow. As a response, in cases of closed ductus arteriosus, pulmonary arterial circulation is partially assured by systemic fetal vessels called major aortopulmonary collateral arteries (MAPCAs) that persist after birth to compensate the impairment of the pulmonary circulation. MAPCAs usually originate from the descending thoracic aorta and probably represent dilated bronchial arteries due to its similar origin and course (Fig. 4a–c) [18]. MAPCAs are essential to assure the pulmonary circulation in such patients until undergoing a corrective surgical procedure, when they are frequently embolized. Hemoptysis may occur, potentially originating from the aneurismal dilation of hypertrophied bronchial vessels, erosion of varicose bronchial vessels into the airway, or rupture of MAPCAs [19, 20]. Hemoptysis may be triggered by infection or co-existing clotting disorders. When it compromises the hemodynamics stability of the patient, embolization of the culprit artery is indicated [17, 20].

Agenesis of the pulmonary artery, also called proximal interruption of the pulmonary artery, is an uncommon developmental anomaly resulting in proximal absence of the pulmonary artery, with preservation of the intrapulmonary vasculature. This variant can occur on either side, more commonly on the right side, with ipsilateral lung hypoplasia [21]. When it occurs, the distal pulmonary arterial branches are usually supplied by hypertrophied bronchial arteries, internal mammary, intercostal, subdiaphragmatic, or coronary arterial collaterals (Fig. 4d).

Clinical presentation varies from asymptomatic incidental finding to massive hemoptysis due to ruptured hypertrophied systemic collaterals, rarely requiring transcatheter embolization [21].

**Fig. 5:**
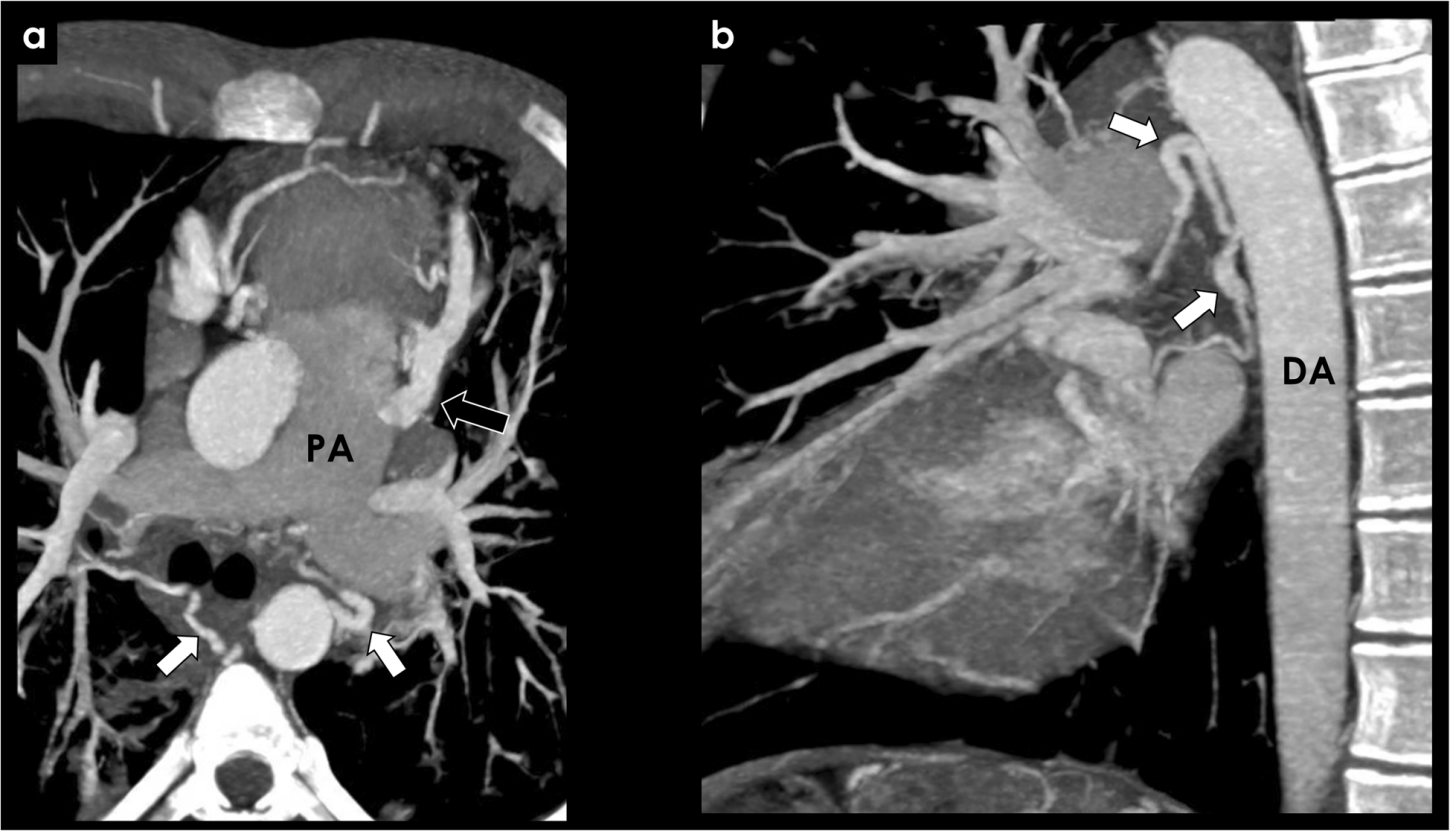
Congenital causes of BAH—ALCAPA syndrome. Patient with an ALCAPA syndrome. CECT showing an anomalous left coronary artery arising from the pulmonary artery (**a**, black arrow) with associated bronchial artery dilatation (white arrows). PA, pulmonary artery; DA, descending aorta

ALCAPA syndrome is a rare congenital disease associated with an anomalous origin of the left coronary artery from the pulmonary artery trunk, leading to a right-to-left shunting. It has a high mortality rate due to myocardial infarction, explained by the diverted flow from the coronary bed to the pulmonary trunk. This is counterbalanced by the development of right coronary to left coronary artery collaterals early in life. It may be asymptomatic, being diagnosed later in childhood. In these patients, bronchial artery dilatation occurs to increase the arterial supply to the left coronary territory (Fig. 5) [22, 23].

### Acquired causes of BAH

There are several acquired diseases that can cause bronchial artery hypertrophy.

**Fig. 6:**
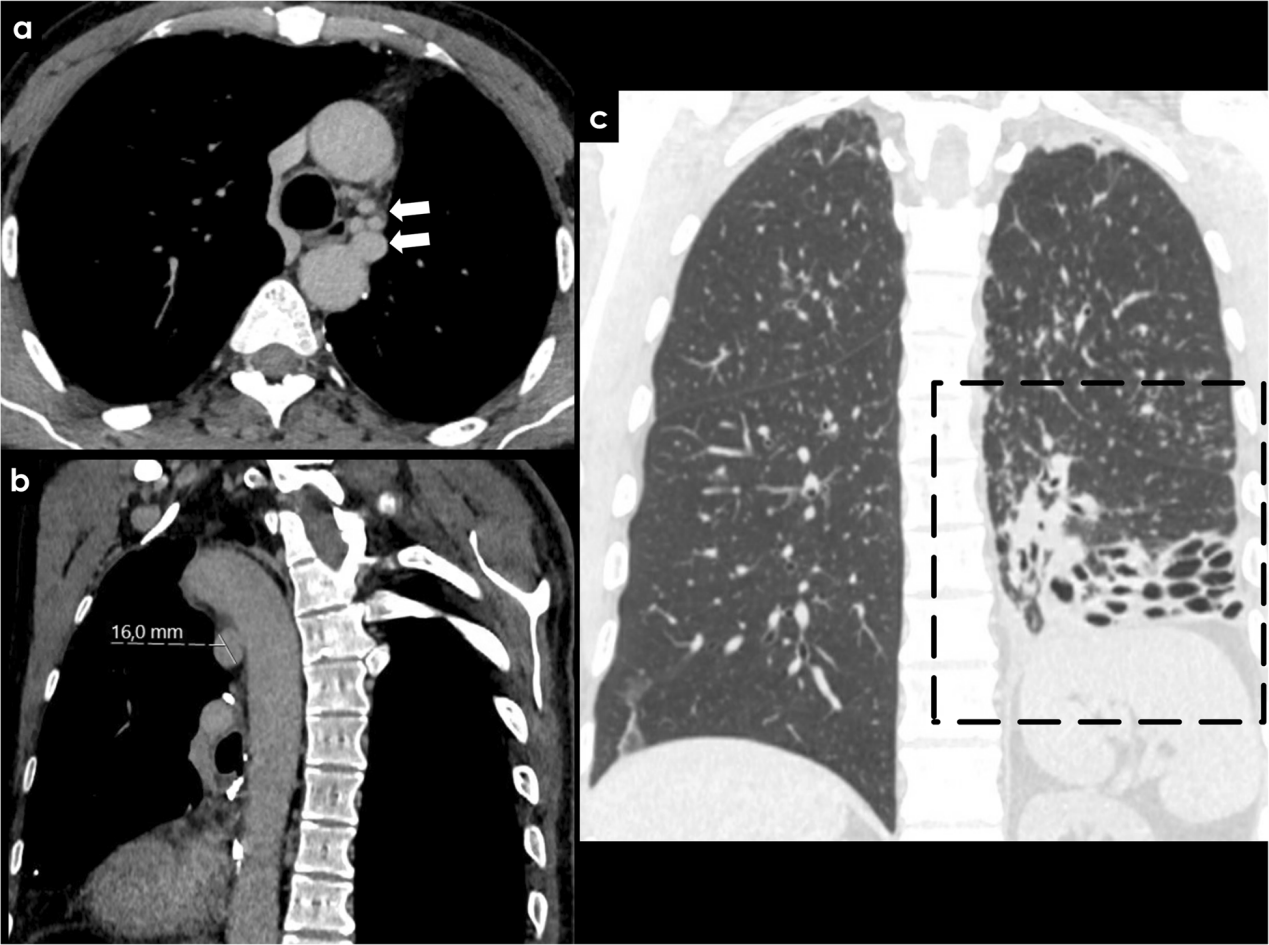
Acquired causes of BAH—bronchiectasis. Patient with a massive left bronchial artery dilatation (**a**, white arrows) reaching 16 mm of caliber (**b**), related to the long-term history of bronchiectasis in the left lower lobe (**c**)

**Fig. 7:**
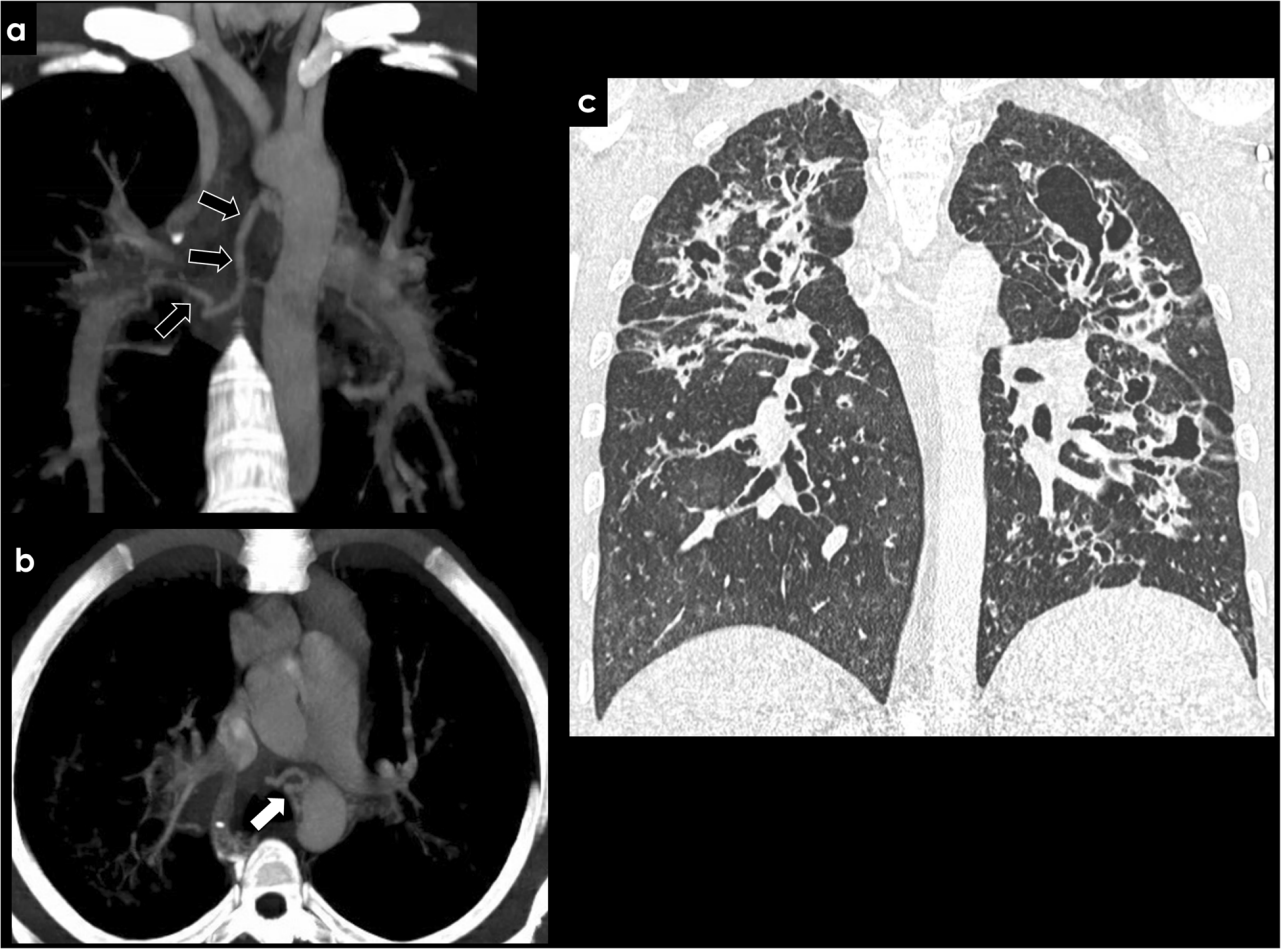
Acquired causes of BAH—cystic fibrosis. Ectopic dilated bronchial artery arising from the distal portion of the aortic arch (**a**, **b**; arrows) in a patient with cystic fibrosis. Typical pulmonary manifestations of cystic fibrosis are well depicted in the image (**c**)

**Fig. 8:**
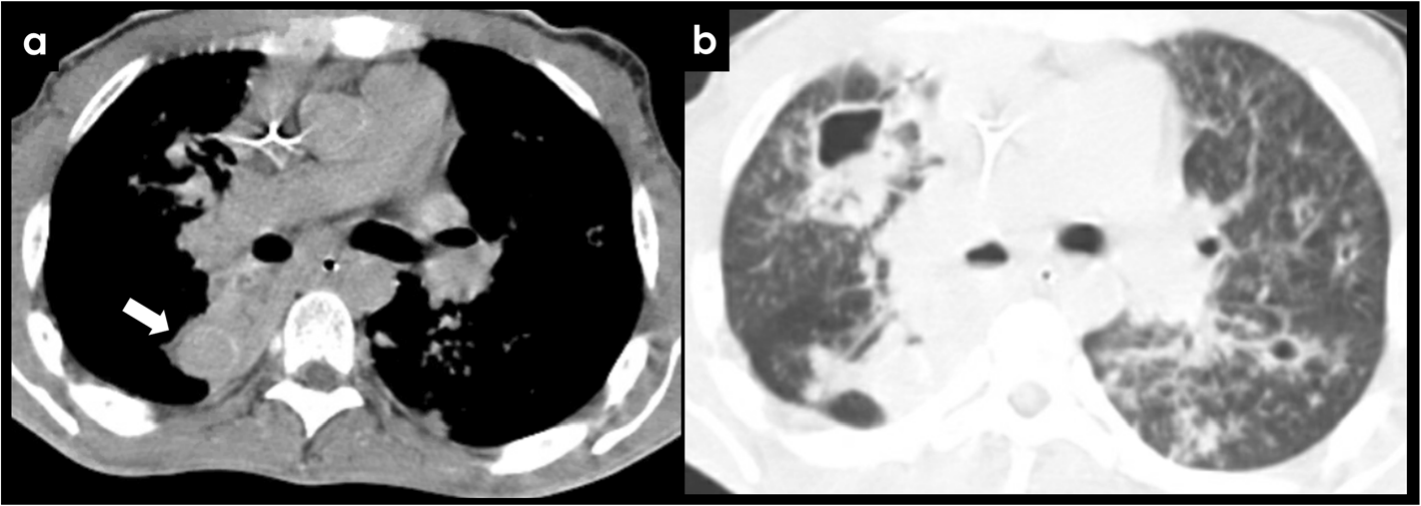
Rasmussen’s aneurysm. Active TB can cause hemoptysis due to rupture of a Rasmussen’s aneurysm (**a**, white arrow), which is a pseudo-aneurysm of the pulmonary artery adjacent to a cavity in patients with pulmonary TB (**b**)

Inflammatory diseases and infection, such as bronchiectasis (Fig. 6), cystic fibrosis (Fig. 7), tuberculosis (TB), or aspergilloma (Fig. 8), may be associated with BAH. The proposed mechanism is complex, probably due to vasculitis with increased neovascularity and microvascular thrombosis of the pulmonary vessels [24, 25]. This process appears to be mediated by angiogenic growth factors, which promote the proliferation and expansion of the bronchial arteries [24].

Bronchiectasis is an acquired disorder characterized by permanent abnormal dilatation and destruction of the bronchial walls. Its pathophysiology involves an infectious insult to the airways, with impaired drainage, airway obstruction, and/or a defect in host defense. There is a multitude of etiologies associated with bronchiectasis, such as cystic fibrosis, cigarette smoking, pulmonary infections, defective host defenses, and foreign-body airway obstruction. Recurrent airway inflammation promotes neovascularity and bronchial artery dilatation, increasing the risk of hemoptysis (Figs. 6 and 7) [25].

Infection is also associated with BAH, and one of the most common agent is Mycobacterium tuberculosis. Nowadays, despite recent medical advances, TB remains a relevant health burden, particularly in developing countries. Historically, hemoptysis was considered almost pathognomonic for pulmonary tuberculosis. Hemoptysis is a potential complication of active or prior TB. Bleeding in active TB can occur in the setting of cavitary or noncavitary disease and usually results from bronchial arterial ulceration, with necrosis of adjacent blood vessels. Rarely, active TB can cause hemoptysis due to rupture of a Rasmussen’s aneurysm, which is a pseudo-aneurysm of the pulmonary artery adjacent to a tuberculous cavity. When it occurs, it may lead to life threatening massive hemoptysis, with associated high mortality rate (Fig. 8) [26].

**Fig. 9:**
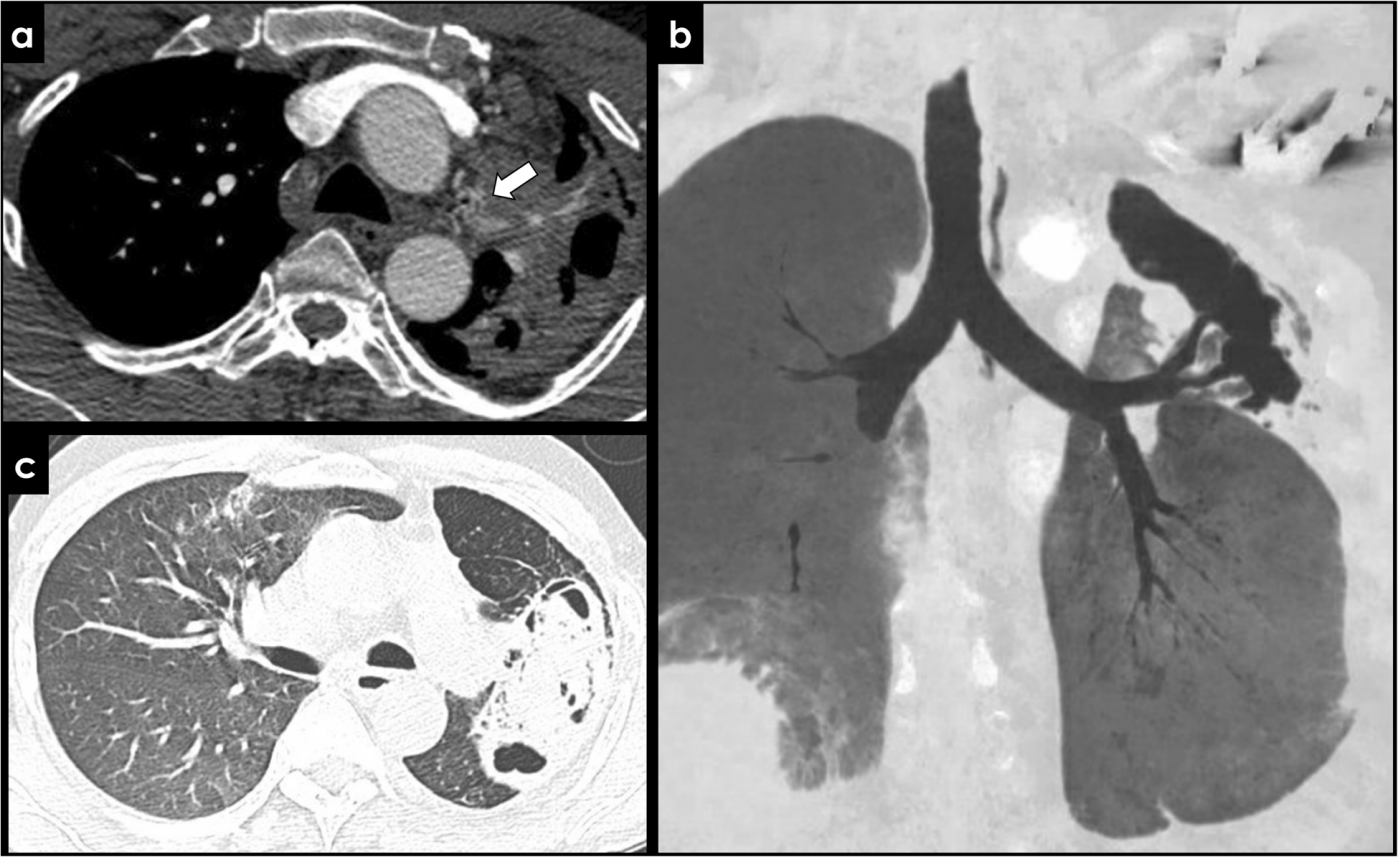
Acquired causes of BAH—tuberculosis and aspergilloma. Left bronchial artery enlargement (**a**, white arrow) in a patient with an aspergilloma complicating a left upper lobe cavity from tuberculosis (**c**). Image **b** represents the same patient 4 years before demonstrating an aerated cavity without signs of fungal colonization

Although strongly associated with active pulmonary TB, hemoptysis can also present following resolution of an acute disease (post-primary TB) due to airway or lung parenchymal distortion with associated vascular changes. This irreversible process leads to distortion and damage of local bronchial vascularization, which becomes hypertrophied, dilated, and exposed, with increased risk of bleeding. Other causes of hemoptysis in the setting of inactive/prior TB include the erosion of a healed calcified lymph node through a bronchial artery, bronchiectasis due to structural lung damage, and fungal colonization of prior lung cavities with *Aspergillus* or other species, leading to hemoptysis due to erosion of adjacent vasculature (Fig. 9) [26, 27].

Other lung infections, particularly lung abscess, can also cause hemoptysis. Bleeding may occur acutely from direct necrosis of lung tissue or in the setting of chronic inflammation, due to rupture of hypertrophied bronchial arteries [27].

**Fig. 10:**
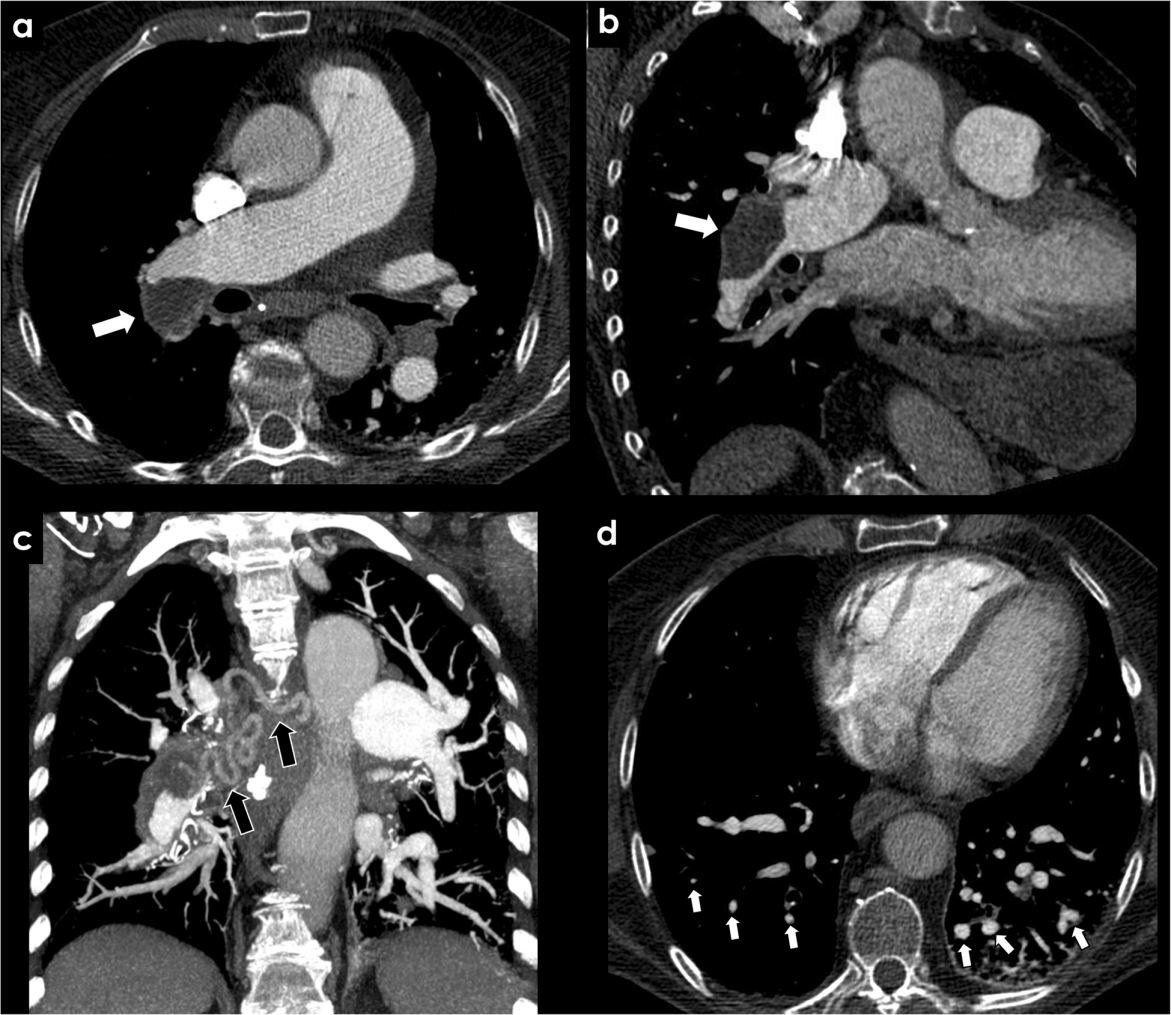
Acquired causes of BAH—chronic thromboembolic disease. CECT findings in a patient with history of chronic thromboembolic disease. A peripheral filling defect with obtuse margins is seen in the right pulmonary artery (**a**, **b**; white arrows), with an asymmetric caliber of distal vessels (**d**, white arrows) and a significant right bronchial artery hypertrophy (**c**, black arrows)

BAH is a potential finding in patients with thromboembolic disease. When it occurs, the presence of dilated bronchial arteries favors chronic disease, and usually coexists on CECT with other vascular signs of chronic thromboembolic disease, such as endoluminal peripheral filling defect with obtuse margins, calcified thrombus, or a web/linear morphology (Fig. 10) [28–30].

Secondary findings include signs of pulmonary hypertension, with increased caliber of the pulmonary arterial trunk, enlargement of the right heart cavities, and parenchymal changes with subpleural linear or wedge-shaped opacities and mosaic attenuation.

Due to chronic pulmonary ischemia, increased incidence of BAH has been reported in chronic thromboembolic disease, potentially associated with moderate-to-massive hemoptysis in 0.1% of the cases [31].

The management of hemoptysis in these patients is challenging due to the ongoing anticoagulant therapy, so even in cases of mild hemoptysis, intervention with embolization should be considered [32].

Pulmonary hypertension is a progressive disease characterized by an elevated pulmonary artery pressure (≥ 25 mmHg at rest). According to its etiology, it is classified as primary, also called pulmonary arterial hypertension (PAH), or secondary, when associated with other diseases like pulmonary thromboembolism, left heart disease, or chronic lung disease.

PAH is primarily a disease of the pulmonary circulation. It is rare, with an estimated incidence of 5–15 cases per one million, being more common in young adults [33]. It affects the small pulmonary muscular arterioles, leading to vasoconstriction, hyperplasia, fibrosis, and thrombosis. It is a severe and progressive disease, with a median survival of 2.8 years if untreated. Common imaging findings of PAH include enlargement of the pulmonary arterial trunk (> 29 mm), narrowing of the distal pulmonary arteries and associated signs of right heart strain. Although rare, BAH is a possible finding in PAH, with a reported incidence of 14%, increasing the risk of hemoptysis [34]. The mechanism of BAH in PAH is not fully understood, being probably related to tissue hypoxia with compensatory hypertrophy of the bronchial arteries. The association of BAH and hemoptysis might be related to the frailty of the hypertrophied bronchial arteries, with more tendency to rupture [35].

The number of dilated bronchial arteries appears to be linked to the increasing severity of the pulmonary arterial hypertension, as a response of the bronchial vasculature to the increasing resistance of flow in the damaged pulmonary vasculature.

Interestingly, patients with non-thromboembolic pulmonary hypertension have a much lower incidence of BAH (10–26%), compared with chronic thromboembolic disease (> 90%). This fact may be helpful in differentiating both diseases [34].

Takayasu arteritis is classified as a large-vessel vasculitis, affecting the aorta, its primary branches, and the pulmonary arteries. It mostly affects young females, usually between 10 and 40 years of age. The pathophysiology is poorly understood, probably related to cell-mediated mechanisms with infiltration of inflammatory cells in the large-vessel walls, leading to vessel narrowing, occlusion, or dilatation of the involved arteries. Imaging findings in the acute phase include large-vessel wall thickening with contrast enhancement, progressing to stenosis, or occlusion in the late phase. Bronchial artery enlargement in Takayasu arteritis is a late finding, with increased risk of hemoptysis, occurring only in patients with marked pulmonary artery stenosis or occlusion, due to chronic hypoxemia of the lung tissue [36, 37].

Fibrosing mediastinitis is an infiltrative benign process with excessive fibrotic reaction in the mediastinum, causing central obstruction of the pulmonary vessels. It is frequently sequelae of a prior local nodal infection, usually histoplasmosis, with associated residual mediastinal calcifications [38]. It can result in compromise of the airway, the esophagus, and the great vessels, with reduction of the pulmonary blood flow when it encases the pulmonary vessels. The process is generally unilateral, with ipsilateral bronchial artery dilatation to the affected site as a compensatory response to lung hypoxemia [39, 40].

Malignancy, in particular lung cancer, is a common cause of hemoptysis in older patients, being responsible for almost 25% of all cases of hemoptysis in the USA [41].

The highest incidence of bleeding occurs with squamous cell carcinoma, followed by adenocarcinoma, small cell, and large cell carcinoma [42]. The possible sources of bleeding include bronchial arterial bleed within the tumor, tumor erosion into the pulmonary artery, or systemic arterial rupture.

The widespread use of bronchial artery embolization in the management of hemoptysis contributed to a decrease of the mortality rate, with a success rate of 77–82% in immediate bleeding control [43, 44].

## Hemoptysis

Hemoptysis, the expectoration of blood from the lower respiratory tract, is a worrying symptom, usually associated with an underlying lung disease. It can vary from a self-limiting mild blood-streaking of sputum to a massive life-threatening hemoptysis, although there is no universal consensus about the blood volume criteria defining the degree of hemoptysis. This is partially explained by the difficulty to quantify the amount of expectorated blood. The term massive hemoptysis is generally reserved for life-threatening situations, associated with airway obstruction, abnormal gas exchange, or hemodynamic instability [45]. As stated before, there is a multitude of diseases that can cause BAH, increasing the risk of hemoptysis.

The most frequent diseases causing hemoptysis are TB, bronchiectasis, and lung cancer [45–47].

Infection, in particular TB, remains one of the main causes of hemoptysis, especially in geographic areas with a higher TB incidence.

According to a study of Agmy et al. from Egypt, which included 341 patients with moderate to severe hemoptysis who underwent bronchial artery embolization, TB (either active or sequelae) was the most common etiology of hemoptysis with an incidence of 57%, followed by bronchiectasis (22%) and aspergilloma (8%) [47]. In terms of absolute frequency, bronchiectasis and malignancy were considered the main causes of hemoptysis in some recently reported studies carried in Greece, South Korea, and Italy, with a lower incidence of TB, probably related to geographical differences [45, 46].

Noninvasive imaging has an important role in patients with hemoptysis, allowing the identification of the underlying cause and potential site of bleeding, improving the treatment approach.

Historically, chest radiography has been useful in the initial imaging approach of patients with hemoptysis. It is readily available, quick, and inexpensive, potentially revealing the side of the process or underlying abnormalities such as cavitation, masses, or chronic lung disease.

However, it has a low sensibility, with reported positive diagnostic yield of only 50%. It identifies the bleeding site in only 46% of cases, in contrast to CECT, which may reveal the bleeding site in 63 to 100% of patients with hemoptysis [46].

**Fig. 11:**
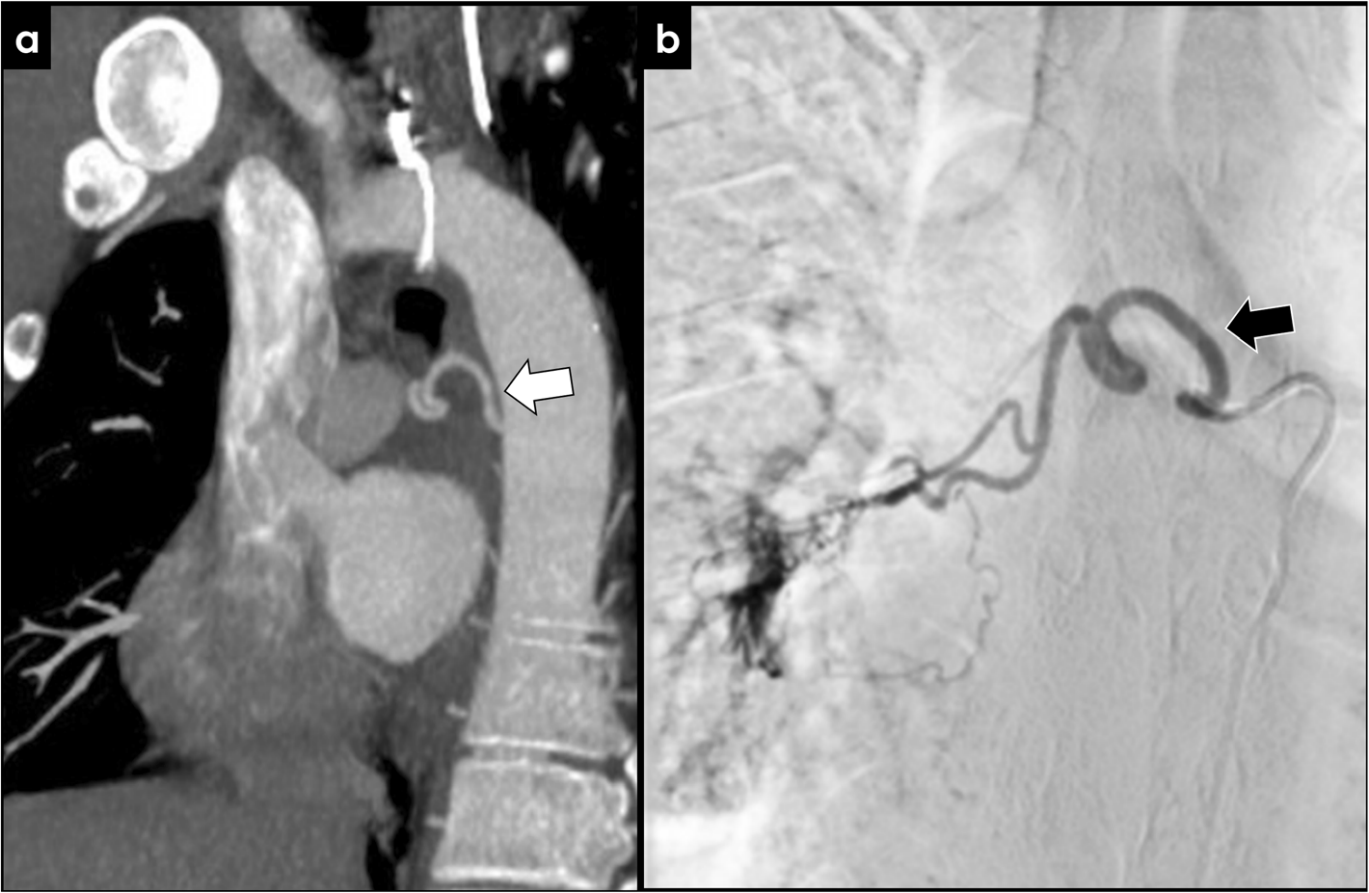
Bronchial artery hypertrophy. Imaging findings of bronchial artery hypertrophy with CECT (**a**, white arrow) and angiography (**b**, black arrow). Hypervascularity and bronchial artery-pulmonary artery shunt are usually well illustrated with angiography (**b**)

CECT is a valuable technique in the clinical context of hemoptysis, allowing a comprehensive analysis of the lung parenchyma, airway, and thoracic vasculature using contrast material. It can depict bronchial artery hypertrophy (most common origin up to 90%) or other potential sites of hemorrhage, underlying diseases and helps to plan the treatment approach (Fig. 11) [48]. When performed before treatment, CECT may reveal the number and origin of the bronchial arteries and possible coexistence of non-bronchial collaterals. A recent study of Lin et al. evaluated the diagnostic performance of CECT in depicting bronchial and non-bronchial systemic arteries in 52 patients with hemoptysis and the feasibility of angiographic embolization. CECT correctly identified 97% of the pathological arteries and facilitated the angiographic approach by revealing its origin, particularly when multiple [49].

CECT is able to identify ectopic vessels amenable for embolization, which potentially reduce recurrence rate of hemoptysis, embolization procedure time, and iatrogenic risks when searching for abnormal vessels in the setting of embolization [50].

**Fig. 12:**
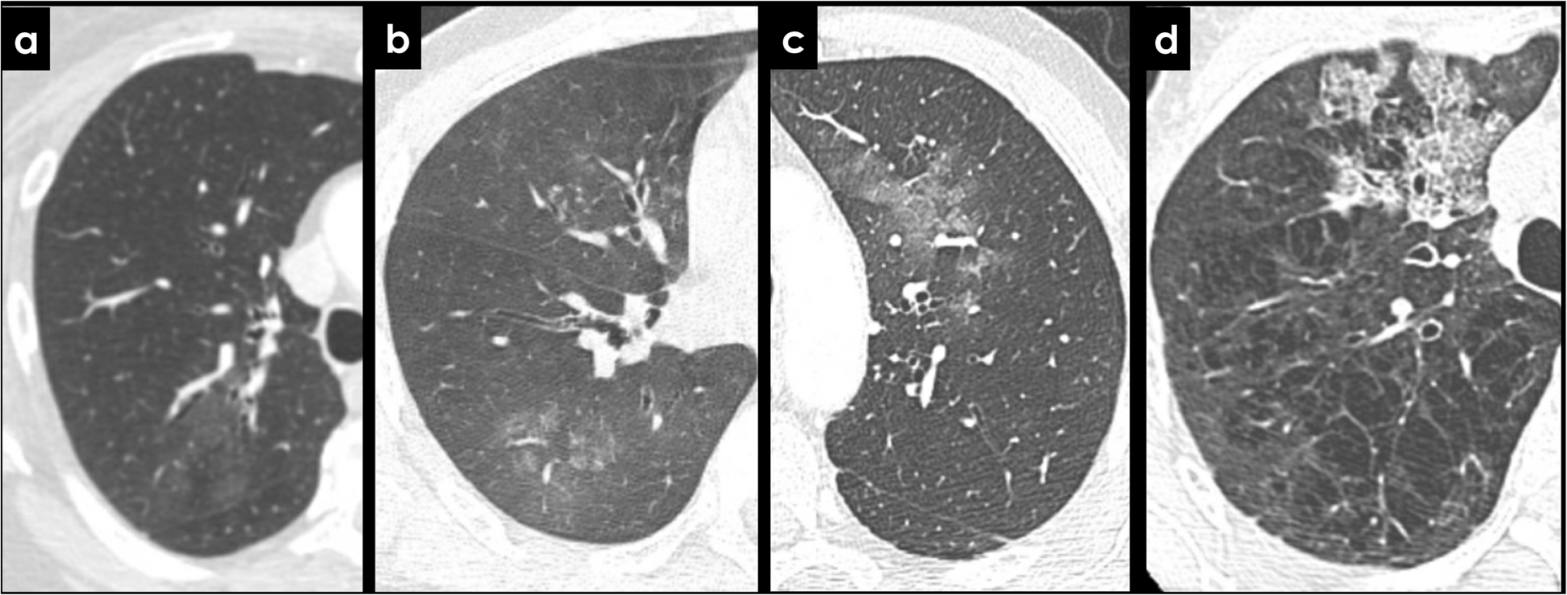
CT findings of pulmonary hemorrhage. Hazy ground-glass infiltrates (**a**, **b**, **c**) representing intra-alveolar hemorrhage in patients with hemoptysis. Also note endobronchial filling in **b** and crazy paving pattern in **d**

Findings of pulmonary hemorrhage on CECT are variable and include airspace opacities varying from ground glass to consolidation, smooth interlobular septal thickening, “crazy-paving” pattern, or centrilobular nodules (Fig. 12). The presence of endobronchial filling material may guide the identification of bleeding site [1].

In summary, CECT has proven its value in the context of hemoptysis, not only allowing the identification of underlying cause and site of bleeding, but also providing a detailed knowledge of the arterial bronchial and non-bronchial anatomy, essential for guiding the endovascular treatment approach.

## Bronchial artery embolization

When BAH occurs, the risk of pulmonary hemorrhage increases, ranging from a small to a potential life-threatening hemoptysis [51].

Bronchial artery embolization is a safe and well-tolerated approach, less invasive than surgery, with proven results in controlling clinically significant hemoptysis [48, 50].

Angiographic approach usually begins with femoral artery cannulation, followed by an aortography of the descending thoracic aorta for identification of bronchial artery and collaterals origin. Ascending aortography and selective subclavian/innominate arteriography are also advocated for identifying apical collaterals. In patients with prior CECT, aortography might be dismissed. Selective catheterization of the bronchial arteries is frequently carried out with 4 or 5 French catheters, facilitated by a reverse-curve catheter configuration. Left main stem bronchus is a common landmark of the level of bronchial artery origin.

**Fig. 13:**
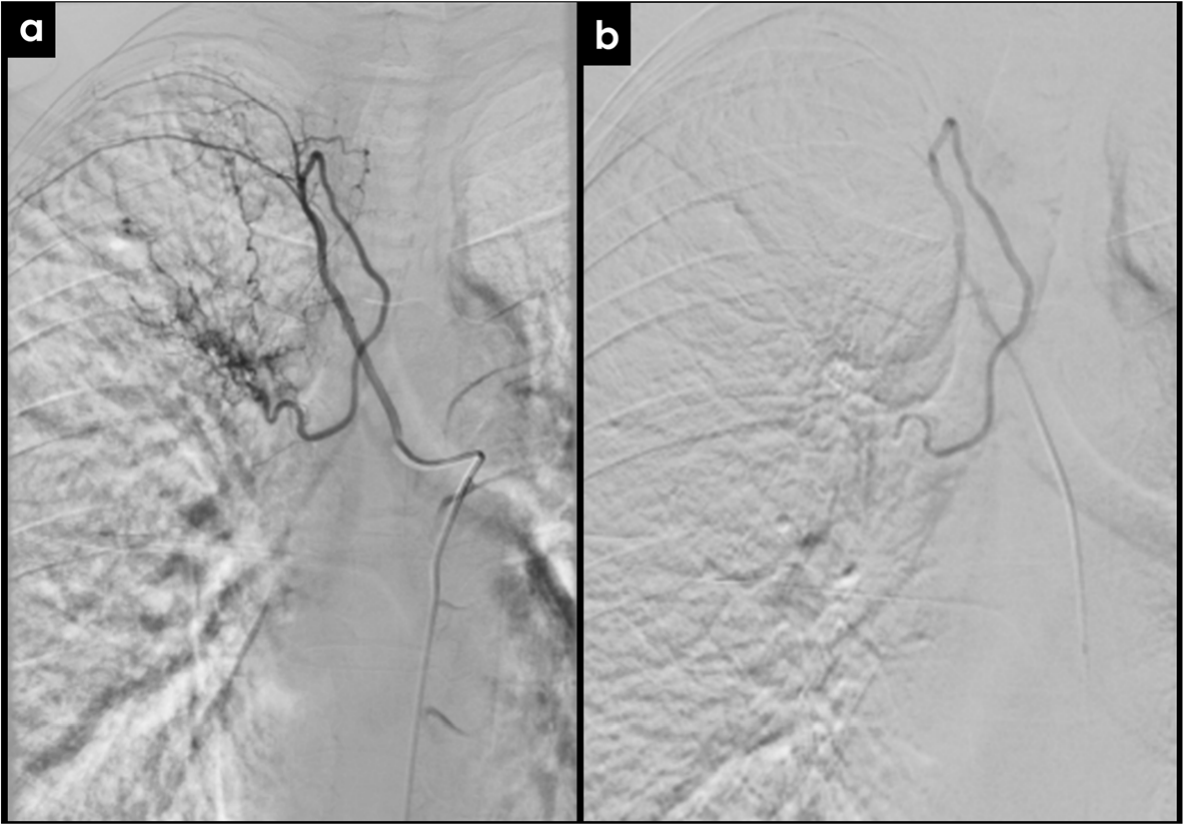
Bronchial artery embolization. Selective catheter angiography before (**a**) and after (**b**) successful right bronchial artery embolization in a patient with hemoptysis in the context of chronic thromboembolic disease

Angiography of the bronchial arteries is usually performed with a flow rate of 2–3 mL/s. After selective bronchial arteriogram, supporting features sensitive for localization of hemoptysis include vascular hypertrophy, tortuosity, hypervascularity, vascular shunting, or extravasation (Fig. 13) [48, 51].

Possible major complications of bronchial artery embolization include transverse myelitis, bronchial infarction, or esophagobronchial fistula. Minor complications are also possible, such as transient chest pain or dysphagia. The most feared complication is spinal cord ischemia related to accidental embolization of a spinal artery, fortunately rare (< 5%) [51].

When performing the embolization, the interventionalist must be cautious with non-target spinal vessels such as the Adamkiewicz artery, the largest anterior medullary branch of the anterior spinal artery. It typically arises from a left posterior intercostal artery at T8–T12 or rarely from T5–T8, with a characteristic hairpin turn or a vertical descent course, and its accidental embolization can have devastating consequences [52]. Therefore, meticulous technique, the use of microcatheters in a coaxial system and distal positioning to the expected location of the spinal artery feeders, can minimize the risk of spinal artery embolization. Superselective embolization is also more effective, improving treatment durability [53].

Polyvinyl alcohol (PVA) particles are commonly used for embolization, ranging from 300 to 700 μm in size. Small particles (< 200 μm) should be avoided because of the increased risk of spinal artery embolization compared with larger particles (> 300 μm) [14, 51].

**Fig. 14:**
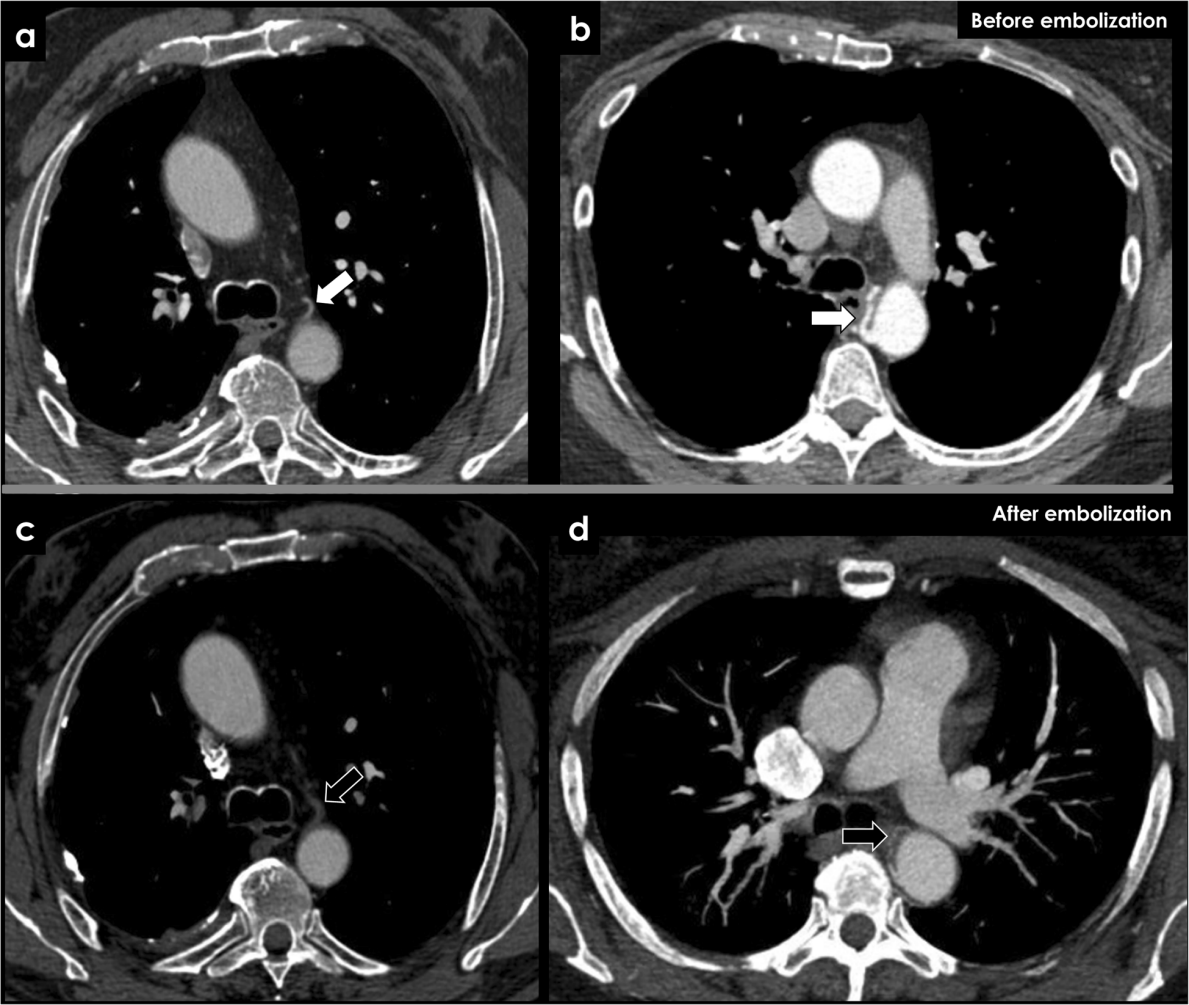
CECT findings of bronchial arteries before and after embolization. After an effective bronchial artery embolization, absence of enhancement (**c**, black arrow) and caliber reduction (**d**, black arrow) are usual CECT findings. We can compare it to the imaging findings before the embolization (**a**, **b**; white arrows)

After embolization of the aimed vascular territory to stasis or near stasis, a reduction in size and enhancement of the bronchial arteries are common findings (Fig. 14).

Bronchial artery embolization has proven to be effective in controlling the potential hazardous hemoptysis, with success rates between 73 and 100% [1, 7, 27, 50]. Nonetheless, long-term recurrence rate of hemoptysis is not neglectable, particularly in patients with TB and aspergilloma, ranging from 10 to 33%. If necessary, in cases of recurrence, re-embolization is adequate in the management of hemoptysis, with similar clinical success compared with the initial embolization procedure [54, 55].

## Conclusion

Radiologists must be familiar with the anatomy of the bronchial arteries. They must be able to recognize when bronchial artery dilatation is present, identify possible underlying diseases, and consider the possibility of bronchial artery embolization in cases of uncontrolled hemoptysis.

Radiologists play a major role in the management of these patients, allowing for the better outcome, particularly in hazardous situations.

## Data Availability

All data and materials presented were from our hospital and daily practice.

## Abbreviations

AA: Ascending aorta
ALCAPA: Anomalous left coronary artery arising from the pulmonary artery
BAH: Bronchial artery hypertrophy
CECT: Contrast-enhanced computer tomography
CT: Computer tomography
DA: Descending aorta
ICA: Intercostal artery
ICBT: Intercostal-bronchial trunk
LBA: Left bronchial artery
MAPCAs: Major aortopulmonary collateral arteries
MIP: Maximum intensity projection
PA: Pulmonary artery
PVA: Polyvinyl alcohol
RBA: Right bronchial artery
TB: Tuberculosis
